# Diagnostic and prognostic multimodal prediction models in Alzheimer's disease: A scoping review

**DOI:** 10.1177/13872877251351630

**Published:** 2025-06-26

**Authors:** Xin Xia, Lukas A Duffner, Christophe Bintener, Angela Bradshaw, Daphné Lamirel, Linus Jönsson

**Affiliations:** 1 Department of Neurobiology, Care Sciences and Society, Section for Neurogeriatrics, Karolinska Institutet, Stockholm, Sweden; 2 Alzheimer Europe, Senningerberg, Luxembourg

**Keywords:** Alzheimer's disease, disease progression, early diagnosis, multimodal, prediction model, scoping review

## Abstract

**Background:**

Multimodal prediction models for Alzheimer's disease (AD) are emerging as promising tools for improving detection and informing prognosis.

**Objective:**

To summarize the predictive objectives, constituting predictors and algorithms, and performance of existing multimodal prediction models.

**Methods:**

We performed a systematic literature search in Medline, Embase, and Web of Science up to January 15, 2024, to identify prediction models covering the full spectrum of AD, from the preclinical stage to subjective cognitive decline (SCD), mild cognitive impairment (MCI), and AD dementia. The predictors, algorithms, and model performance of prediction models were summarized narratively by their predictive objectives. The review protocol was registered with the Open Science Framework (osf.io/zkw6g).

**Results:**

Predicting the future progression from MCI to AD dementia was the most common objective of prediction models for AD. The second most common objective was to classify AD stages (SCD versus MCI versus AD dementia), followed by detecting the presence of amyloid, tau, or neurodegeneration. More than half of the prediction models reported an area under the receiver operating characteristic curve exceeding 0.8 and an accuracy exceeding 70%. However, 66.7% of the prediction models were developed using data from the ADNI study, and only 10.1% of the models went through external validation.

**Conclusions:**

Existing multimodal prediction models have mainly focused on the prediction of current or future AD stages and reported good performance. However, these models need to be validated using data other than the data used for model training before being considered for practical applications.

## Introduction

Alzheimer's disease (AD) is the most common cause of dementia and a significant contributor to morbidity and mortality in older age.^1^ There have been important advances in disease-modifying treatments for AD in recent years.^2,3^ These treatments have been shown to benefit people who are in the early stages of AD, including mild cognitive impairment (MCI) due to AD and mild AD dementia.^2,3^ The advancement of disease-modifying therapies for AD has profound implications for the diagnosis and treatment of AD, underscoring the crucial significance of timely detection and close monitoring of disease progression.

There is a substantial amount of research investigating the value of various types of patient information (e.g., neuropsychological tests, neuroimaging biomarkers) in informing the diagnosis and prognosis of AD.^4–9^ There have been ongoing efforts to construct prediction models for AD to improve the prediction of dementia status and inform prognosis.^10–12^ Among them, multimodal prediction models, which consider at least two data modalities, such as cognitive tests combined with fluid biomarkers, have great potential to be used to assist clinical decision-making in dementia diagnosis and management and to be integrated into computerized clinical decision support systems.^11,13^ The present scoping review focuses on multimodal prediction models instead of monomodal prediction models because (1) there have been literature reviews summarizing the predictive value of single data modalities, and (2) multimodal data reflects the information from people with AD that is usually collected in clinical settings and the complexity of AD better than monomodal data.^4–9^

Previous literature reviews on prediction models have mainly focused on differentiating AD dementia from healthy controls or predicting progression from MCI to AD dementia.^10,11,14^ However, previous prediction models have also aimed to predict other relevant outcomes, such as the prognosis of SCD and amyloid positivity. Despite this, no review has systematically summarized multimodal prediction models for AD without focusing solely on specific prediction objectives. Therefore, this literature review aims to provide a comprehensive summary of existing multimodal prediction models in AD, covering the full range of possible objectives and outcomes. The breadth of this rapidly evolving research field warranted a scoping approach to map the extent, range and nature of the existing literature and determine possible gaps. We therefore decided to conduct a scoping review.

## Methods

The literature review was conducted following the Arksey and O’Malley framework for scoping reviews, which includes five major steps: defining research questions, identifying potentially relevant studies, selecting relevant studies, extracting data from included studies, and summarizing the data and reporting the results.^15^ The literature review protocol was registered with the Open Science Framework (osf.io/zkw6g). We reported the results following the Preferred Reporting Items for Systematic reviews and Meta-Analyses extension for Scoping Reviews (PRISMA-ScR) Checklist.

### Eligibility criteria

The studies included in the literature review met the following criteria: (1) they were conducted in human subjects, excluding rodent studies, (2) they used cross-sectional, case-control, or longitudinal study designs, including secondary analyses of clinical trial data, (3) the studies focused on prediction models trained on data from individuals with suspected AD symptoms, (4) the prediction models incorporated two or more data modalities, with studies using only neuroimaging modalities (e.g., MRI and PET) considered single-modality and therefore excluded, and (5) they were original investigations published in peer-reviewed journals.

Exclusion criteria for the literature review were: (1) records that were editorials, commentaries, reviews, or viewpoints, (2) studies that did not report model performance measures, and (3) retracted studies.

### Information source and search strategy

We conducted a systematic literature search of literature, until January 15, 2024, with no restriction on the inception date, in Medline, Embase, and Web of Science.

A Medline search strategy was first developed by two authors (XX and LJ) by combining three sets of MeSH terms and free texts that indicated (1) AD and dementia, (2) disease diagnosis and prognosis, and (3) multimodal prediction models. A librarian from the Karolinska Institutet University Library revised and translated the Medline search strategy to search strategies in Embase and Web of Science. In addition, the librarian de-duplicated the publications using standard methods.^16^ We restricted our search to publications in English. The full search strategy is reported in Supplemental Material 1.

### Study selection

We adopted a semantic analysis-assisted approach for screening the titles and abstracts, considering the large amount of literature for screening. This approach consisted of 3 major steps. In Step 1, 111 benchmark references for semantic analysis were identified by a researcher (XX) by reviewing titles and abstracts of 1000 randomly selected articles. In Step 2, a semantic analysis was performed on the titles and abstracts of the remaining articles with the benchmark articles identified in Step 1. The remaining articles were ranked from the largest to the smallest values of the similarity measure estimated from the semantic analysis. Then, the researcher reviewed the titles and abstracts by the rank of the similarity measure until the titles and abstracts no longer appeared to be relevant. Lastly, the researcher screened only the titles of the remaining references. In Step 3, two reviewers from the literature review team conducted a full-text review of each article that passed the title and abstract screening. In addition, references of included articles were scrutinized for eligible studies.

The semantic analysis was performed using “SentenceTransformers” in Python (version 3.10.11). Any ambiguities or uncertainties during the study selection process were resolved through discussions among the authors and in consultation with the senior researcher (LJ).

### Data extraction

A data extraction template was created by a researcher (XX). The data extraction template included identification information of the studies (i.e., author, year, title), basic characteristics of the studies (e.g., the predictive objective, the study setting, participant demographics), and information about the multimodal prediction models. The latter included the predictive factors that were included in the prediction models (hereafter referred to as predictors), the statistical models or machine learning algorithms constituting the prediction models, the predicted outcomes and their definitions, and model performance and validation. It was common for studies to evaluate multiple candidate prediction models, in which case we extracted data from the prediction models with the best performance based on area under the receiver operating characteristic curve (AUC) or accuracy if AUC was not reported.

Each of the authors involved in the data extraction process (XX, LAD, CB, AB, DL) had at least one trial of extracting relevant information from five studies using the data extraction template. Any ambiguity or problems regarding the data extraction process were resolved through discussions in group meetings. A secondary review of the extracted data was conducted by a different reviewer than the one who initially extracted the data.

### Data synthesis

The data from the prediction models in the included studies were synthesized narratively and charted based on the outcomes the studies aimed to predict. Notably, a single study may have multiple predictive objectives, such as distinguishing AD from Lewy body dementia and differentiating AD from vascular dementia, each of which was treated as a separate prediction model.

We also quantitatively summarized the demographic information (e.g., mean age, the proportion of females) of the included studies and mapped the multimodal prediction models and their performances. We summarized four commonly reported model performance metrics: AUC, accuracy, sensitivity, and specificity.

Lastly, we summarized the prediction models that demonstrated good performance based on the AUC and accuracy parameters. Good performance was defined as an AUC > 0.9 for models predicting MCI progression to AD or an AUC > 0.8 for other predictive objectives, given that there were significantly more prediction models for MCI progression to AD than for other predictive purposes. For models lacking AUC data, which is common in multi-class classification tasks such as classifying individuals into having SCD, MCI, or AD dementia, an accuracy exceeding the minimum accuracy of models with the same predictive objective and high AUC was also considered good performance. For this summary, we excluded prediction models that did not report basic demographic information, were trained on less than 100 persons, or included tests that were key criteria for defining the outcome as predictors, considering the risk of inadequate reporting and overestimated performance of these models.

The quantitative analysis and data visualization were performed using R statistical software (version 4.3.3).

## Results

**Figure 1.**
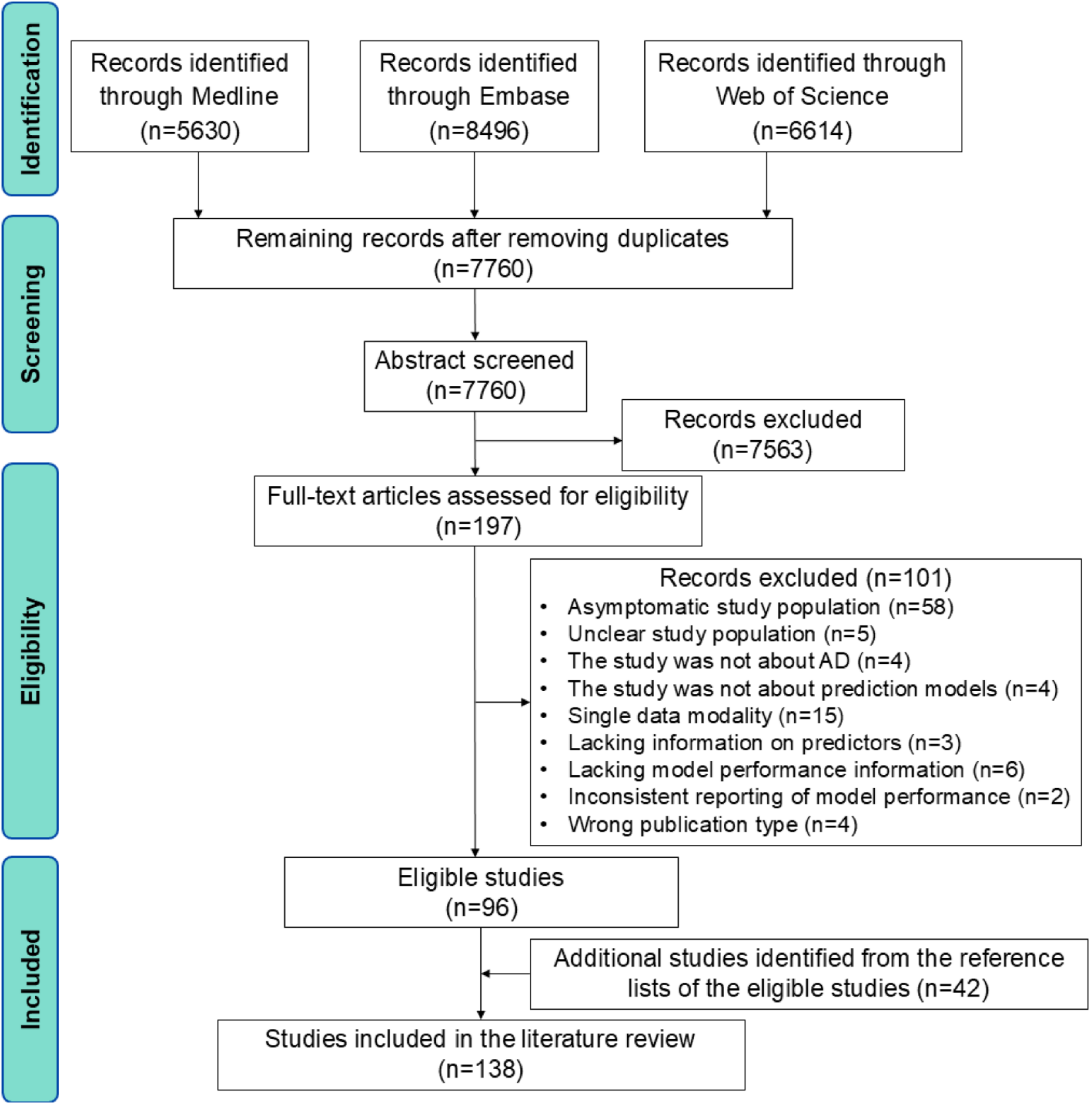
Flowchart of study selection. AD: Alzheimer's disease.

We identified 5630 studies from Medline, 8496 studies from Embase, and 6614 studies from Web of Science. After removing duplicates, 7563 studies were further excluded after the review of titles and abstracts, resulting in 197 studies eligible for full-text review. Following full-text review, 101 ineligible studies were excluded. The remaining 96 studies, along with 42 additional studies identified from their reference lists, were included in the literature review (Figure 1). The list of included studies can be found in Supplemental Material 2.

### Characteristics of the included studies

We identified six main predictive objectives from the included studies: (1) classifying the stages of AD (SCD versus MCI versus AD dementia), (2) detecting the presence of amyloid, tau, or neurodegeneration, (3) differentiating AD dementia from other types of dementia, (4) predicting SCD conversion to MCI or AD dementia, (5) predicting MCI conversion to AD dementia, and (6) predicting the rate of cognitive decline (Table 1). More than half of the included studies aimed to develop multimodal prediction models for predicting the likelihood of developing AD dementia in people with MCI (N = 89; 64.5%). The second most common goal of the multimodal prediction models was to predict the stage of AD (N = 26; 18.8%), followed by the prediction of the presence of amyloid, tau, or neurodegeneration (N = 19; 13.8%), and differentiating AD dementia from other types of dementia (N = 13; 9.4%). Less common goals of existing multimodal prediction models included predicting conversion from SCD to MCI or to AD dementia (N = 5, 3.6%) and the rate of cognitive decline (N = 6; 4.3%). Other predictive objectives included predicting the conversion from the absence of amyloid neuropathology to the presence of cerebral amyloid neuropathology, predicting years to symptom onset of AD, and predicting functional decline.^17–19^

**Table 1.** Characteristics of the included studies.

Characteristics	Number of studies (N = 138)
**Predictive objective^a^**	
MCI progression to AD dementia	89 (64.5%)
SCD versus MCI versus AD dementia	26 (18.8%)
Detecting the presence of amyloid, tau, or neurodegeneration	19 (13.8%)
Differentiating AD dementia from other types of dementia	13 (9.4%)
Predicting decline in cognitive test scores	6 (4.3%)
SCD progression to MCI or AD dementia	5 (3.6%)
Other predictive objectives	3 (2.2%)
**Mean age**	
<60 years	1 (0.7%)
60–70 years	14 (10.1%)
70–80 years	105 (76.1%)
**Percentage of females**	
<30%	5 (3.6%)
30%-40%	31 (22.5%)
40%-50%	57 (41.3%)
50%-60%	21 (15.2%)
≥60%	7 (5.1%)
**Study based on ADNI**	
No	46 (33.3%)
Yes	92 (66.7%)
**External validation**	
Yes	13 (9.4%)
No	123 (89.1%)
Not applicable^b^	1 (0.7%)
Unclear	1 (0.7%)

Notes: There are 18 studies missing age information and 17 studies missing numbers of females.

^a^ The total number of predictive objectives is bigger than the number of studies because some studies have multiple predictive objectives.

^b^ The study validated a previous prediction model.

AD: Alzheimer's disease; ADNI: Alzheimer's Disease Neuroimaging Initiative; MCI: mild cognitive impairment; SCD: subjective cognitive decline.

More than 70% of the included studies consisted of a study population whose mean age was above 70 years old, and females were often underrepresented (Table 1). Among the included studies, 92 (66.7%) were based on the Alzheimer's Disease Neuroimaging Initiative (ADNI), which recruited participants from communities in the United States. Only 14 (10.1%) studies conducted external validation for the prediction models.

In addition to the ADNI-based studies and 9 other studies conducted in the United States, 14 (10.1%) studies were conducted in Asian populations, 15 studies (10.9%) in European populations, 1 in Brazil, 1 in Canada, and 6 were multinational. Most of the included studies had relatively small sample sizes, with 21 (15.2%) studies having less than 100 participants, 55 (39.9%) studies having less than 200 participants, and 102 (73.9%) studies having less than 500 participants.

### Predictors and statistical models or algorithms of the prediction models

A summary of predictors included in the multimodal prediction models is shown in Table 2. Included predictors largely aligned with important factors for the diagnosis of AD, such as medical history, cognitive assessment, and brain imaging. However, computerized tomography, which is often performed during the diagnostic process, has not been considered in the identified multimodal prediction models.^20^

**Table 2.** Summary of predictors included in more than one multimodal prediction models.

Cognitive test scores	**Overall cognitive function:** MMSE, ADAS-Cog, CDR, CDR-SB, clock-drawing test, MoCA, CERAD, Addenbrooke's Cognitive Examination, CAMCOG
	**Domain-specific cognitive tests:** RAVLT, TMT-A, TMT-B, Logical Memory, animal category fluency test, BNT, the Stroop test, Wechsler Memory Scale, ADNI summary tests of memory, ADNI summary tests of executive function, RCFT
Functional tests	FAQ, ADL, IADL
Neuroimaging predictors	**MRI features:**
	T1-weighted MRI (whole brain volume, grey matter volume, white matter volume, hippocampal volume, entorhinal cortex volume, ventricular volume, cortical atrophy) DTI T2-weighted MRI/FLAIR (white matter hyperintensity)
	**PET features:** FDG-PET Amyloid PET
Fluid biomarkers	**CSF biomarkers:** Aβ_42_, t-tau, p-tau, p-tau/Aβ_42_, t-tau/Aβ_42_, neurogranin
	**Blood-based biomarkers:** Aβ_40_, Aβ_42_, p-tau181, Aβ_42_/Aβ_40_
Genetic predictors	*APOE* genotype, *APOE* ε4 allele carriership, number of *APOE* ε4 alleles, polygenic risk score, AD-associated SNPs
Other clinical predictors	**Medical history:** diabetes, hypertension, dyslipidemia, cardiovascular diseases, cerebrovascular diseases, sleep disorders, mental disorders, psychiatric disorders, family history of cognitive impairment or AD
	**Depression test:** GDS
	**Neuropsychiatric symptoms:** NPI
	**Other:** alcohol use disorder, tobacco use, body mass index, cognitive fluctuations, Hachinski ischemic score, hallucinations
Non-clinical predictors	Age, sex, education, marital status, race

AD: Alzheimer's disease; ADAS-Cog: Alzheimer's Disease Assessment Scale-cognitive subscale; ADL: Activities of daily living; ADNI: Alzheimer's Disease Neuroimaging Initiative; BNT: Boston Naming Test; CAMCOG: Cambridge Cognitive Examination; CDR: Clinical Dementia Rating; CDR-SB: Clinical Dementia Rating-Sum of Boxes; CERAD: Consortium to Establish a Registry for Alzheimer's Disease test; CSF: cerebrospinal fluid; DTI: Diffusion-tensor imaging; FAQ: Functional activities questionnaire; FDG-PET: fluorodeoxyglucose-positron emission tomography; FLAIR: Fluid attenuated inversion recovery; GDS: Geriatric Depression Scalel; IADL: Instrumental activities of daily living; MMSE: Mini-Mental State Examination; MoCA: Montreal Cognitive Assessment; MRI: Magnetic resonance imaging; NPI: Neuropsychiatric Inventory; PET: positron emission tomography; RAVLT: Rey Auditory Verbal Learning Test; RCFT: Rey Complex Figure Test and Recognition Trial; SNP: single-nucleotide polymorphism; TMT: Trail Making Test.

Notes: Brain regions frequently investigated in different neuroimaging modalities included hippocampus, entorhinal cortex, inferior temporal, cingulate cortex, middle temporal, fusiform, precuneus, inferior parietal, amygdala, medial temporal, insula, parahippocampal, superior parietal, medial orbitofrontal cortex, superior frontal, and superior temporal regions.

**Figure 2.**
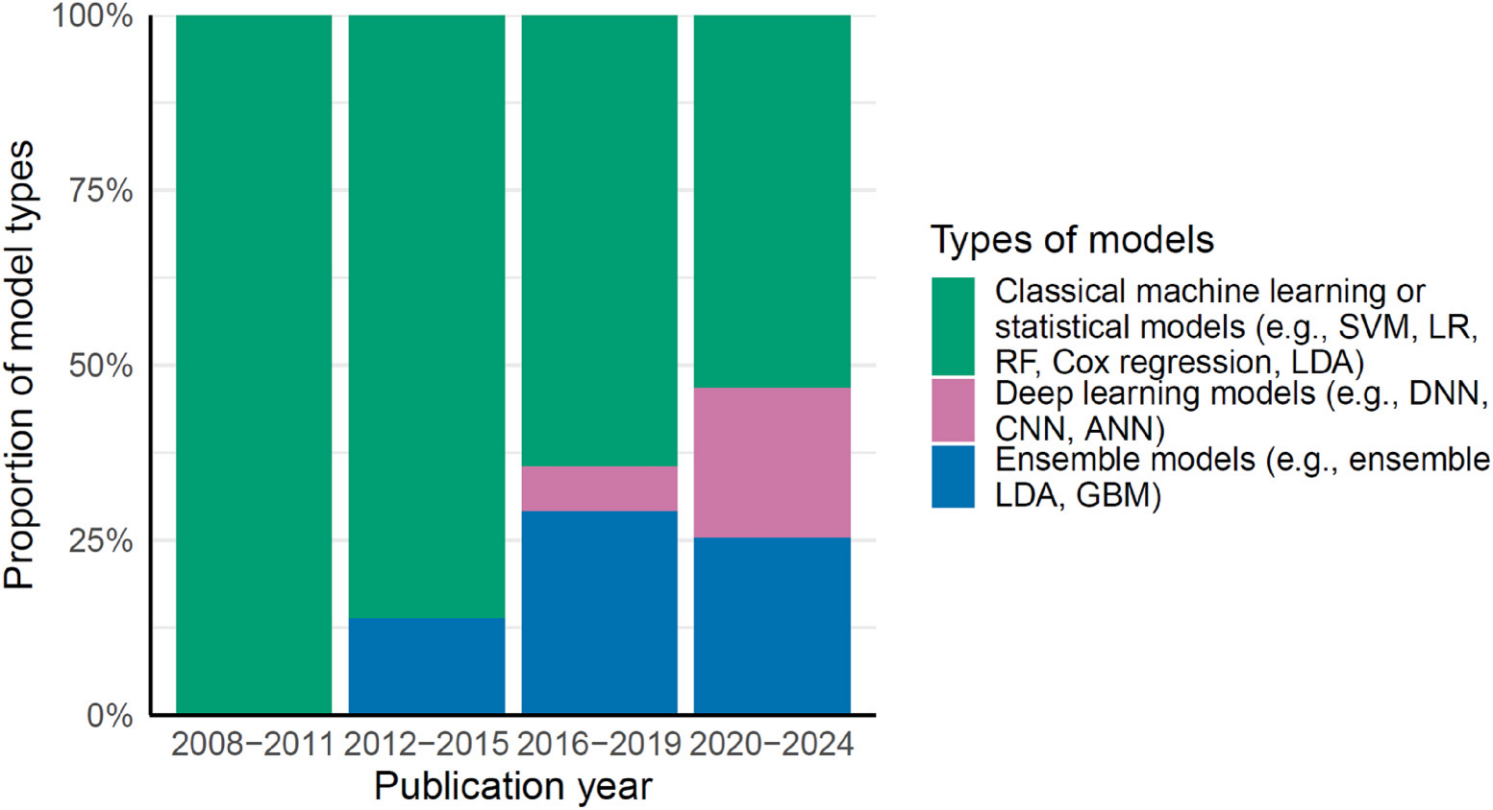
The proportion of statistical models or algorithms used by year. ANN: Artificial Neural Network; CNN: Convolutional Neural Network; DNN: Deep neural networks; GBM: Gradient Boosting Machine; LDA: Linear discriminant analysis; LR: logistic regression; RF: Random Forest; SVM: Support Vector Machines.

The proportion of prediction models using deep learning algorithms, such as deep neural networks, has increased over the years, while the use of classical machine learning or statistical models, such as support vector machines and logistic regression, has declined (Figure 2). There were also studies that applied machine learning algorithms to process neuroimaging data before including them in the final prediction models. For example, a machine learning-derived AD-resemblance atrophy index, an algorithm-computed hippocampal volumetric integrity, and brain atrophy patterns derived from unsupervised clustering have been included as predictors in several prediction models.^21–23^

### Performance of the prediction models

**Figure 3.**
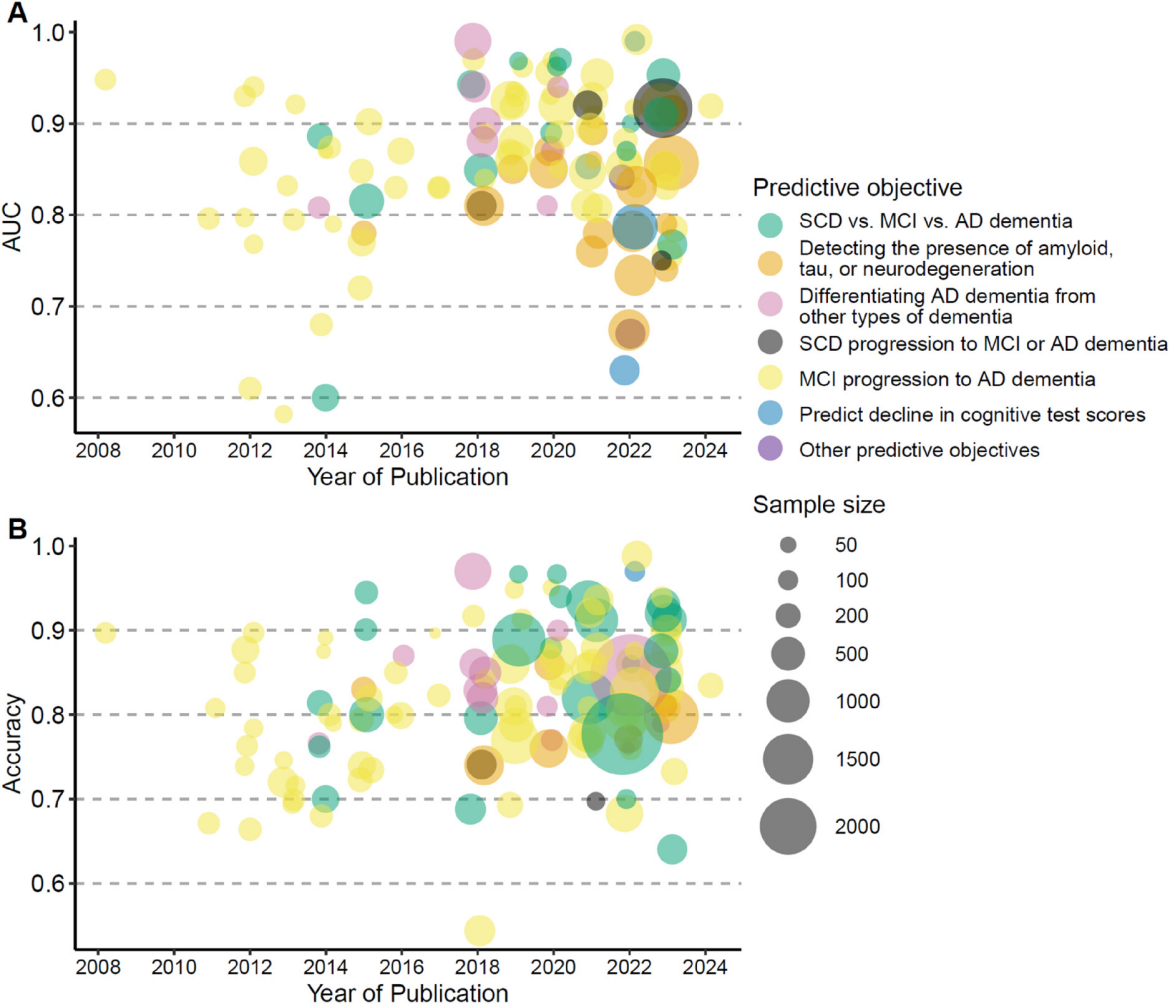
Performance of prediction models. (A) AUC of the prediction models. (B) Accuracy of the prediction models. AD: Alzheimer's disease; AUC: Area under the Receiver Operating Characteristic Curve; MCI: mild cognitive impairment; SCD: subjective cognitive decline.

More than half of the prediction models reported an AUC exceeding 0.8 and an accuracy exceeding 70% (Figure 3), along with sensitivity and specificity above 70% (Supplemental Material 3, Supplemental Figure 1). Prediction models for MCI conversion to AD dementia, classifying AD stages, detecting amyloid, tau, or neurodegeneration, and differential diagnosis generally reported good accuracies. The more recent prediction models were generally built on data from more participants and reported better model performance than older prediction models (Figure 3 and Supplemental Material 4, Supplemental Table 1).

In studies that performed external validation of prediction models, eight out of eleven studies reported a lower AUC in external validation datasets compared to development datasets (Supplemental Material 3, Supplemental Figure 2). The accuracy was lower in the external validation datasets than in the development datasets in half of the six studies that reported accuracy.

### Performance of prognostic models by time horizons

**Figure 4.**
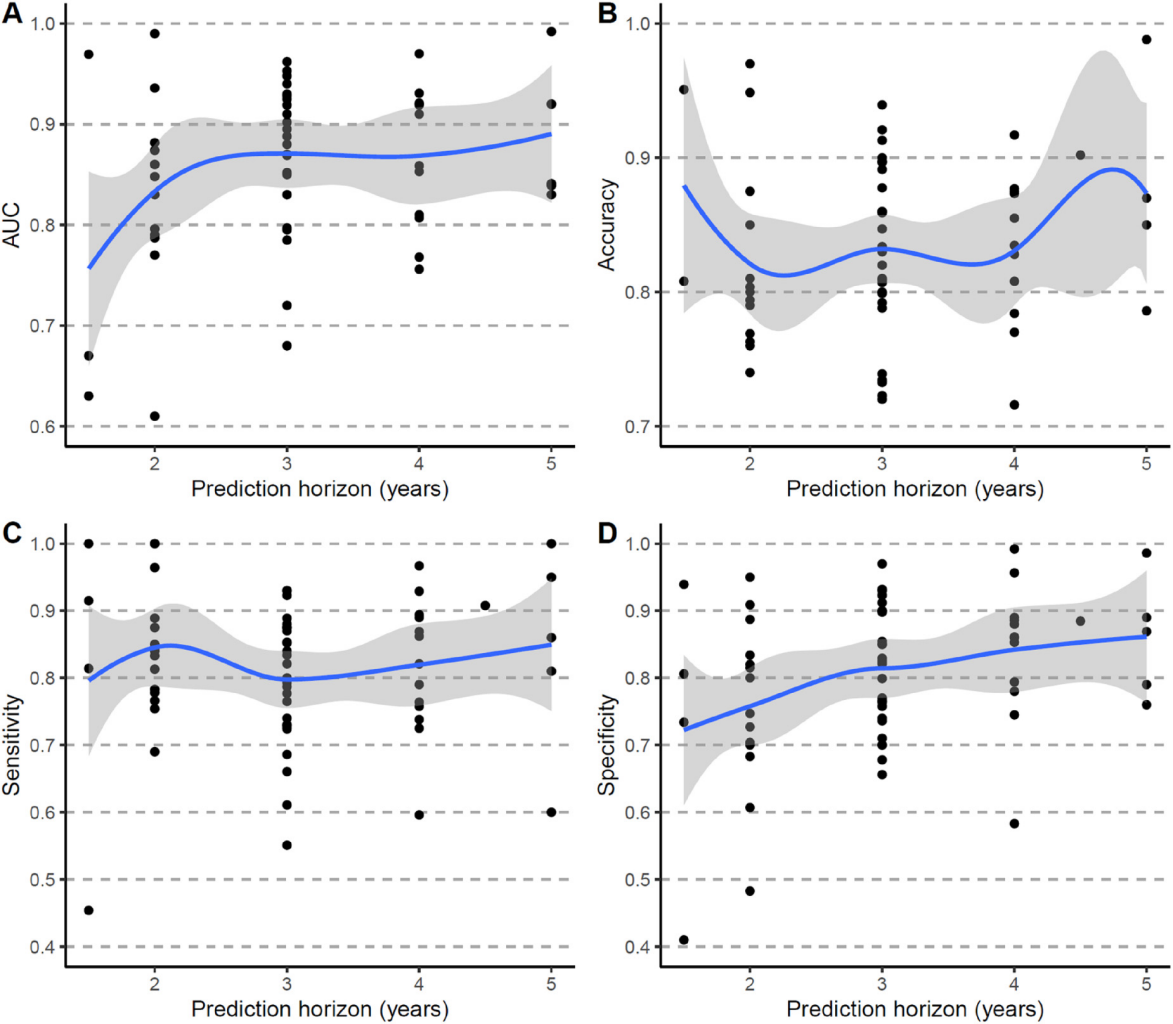
Performance of prognostic models by prediction horizon across studies. (A) AUC. (B) Accuracy. (C) Sensitivity. (D) Specificity. AUC: Area under the Receiver Operating Characteristic Curve.

**Figure 5.**
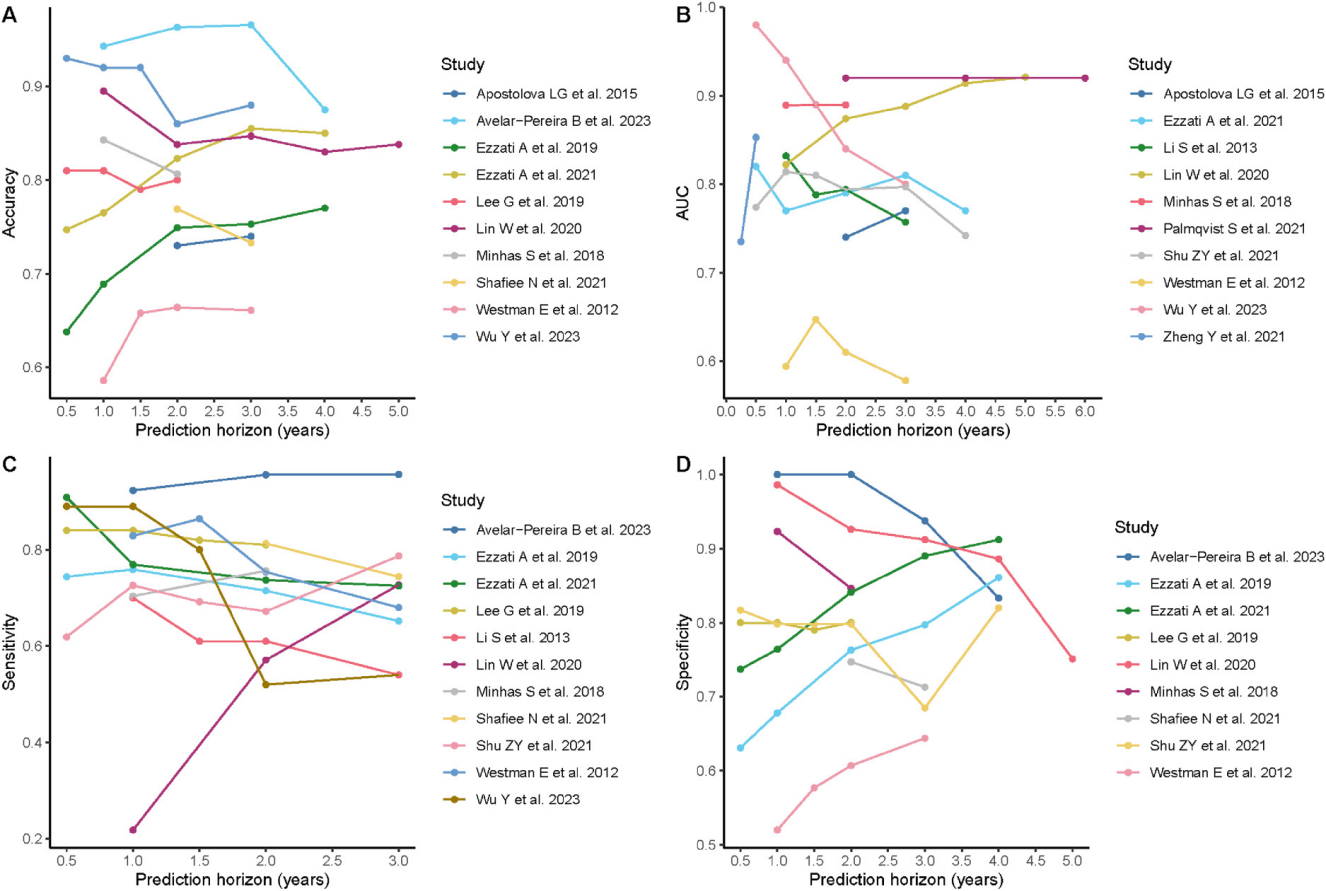
Performance of prognostic models by prediction horizon within studies. AUC: Area under the Receiver Operating Characteristic Curve.

For prognostic models, such as those predicting future progression from MCI to AD dementia, the prediction horizon varied across studies, with two to four years being the most common. Model performance fluctuated with different prediction horizons across studies (Figure 4). Additionally, studies that evaluated their models under multiple prediction horizons also showed variations in performance within the same study as shown in Figure 5 and illustrated in previous studies.^24–27^

### Overview of potentially well-performing prediction models

Table 3 summarizes the predictors and statistical models or algorithms for potentially high-performing prediction models, defined by AUC or accuracy as outlined in the methods section above. It is worth noting that before ranking the performance of the prediction models, we excluded 79 models due to concerns regarding inadequate reporting and overestimated model performance. These models included those that did not report basic demographic information, were trained on small datasets, or used predictors that were important for defining the outcomes of interest.

**Table 3.** Summary of well-performing prediction models.

Predictive objective	Summary
SCD versus MCI versus AD dementia^a,^ ^28–33^	**Predictors:** MMSE, CDR-SB, clock drawing, RAVLT, digit span backwards, TMT-A, TMT-B, category fluency, logical memory, structural MRI volumetric measurements, CSF (Aβ_42_, p-tau, t-tau), *APOE* genotype, age, sex, education
	**Statistical models or algorithms:** Discriminant classification analysis, ELM, LR, LDA, multimodal data fusion model with self-attentive mechanism
	**Model performance:** AUC = 0.815–0.943, Accuracy = 0.8–0.945, Sensitivity = 0.333–0.946, Specificity = 0.73–0.973
Detecting the presence of amyloid, tau, or neurodegeneration^b,^ ^34–41^	**Predictors:** MMSE, ADAS-Cog, CDR-SB, clock drawing, logical memory, BNT, the Stroop test, RAVLT, category fluency, MRI volumetric measurements, CSF (Aβ_42_, p-tau, t-tau), blood (Aβ_40_, p-tau181), *APOE* genotype, age, sex, comorbidities (e.g., diabetes)
	**Statistical models or algorithms:** Ensemble LDA, GBM, LASSO regression, LR, SVM, XGBoost, RF
	**Model performance:** AUC = 0.81–0.914, Accuracy = 0.74–0.859, Sensitivity = 0.71–0.911, Specificity = 0.77–0.903
Differentiate AD dementia from other types of dementia^42–47^	**Predictors:** MMSE, the Stroop test, TMT-A, TMT-B, RAVLT, animal category fluency test, BNT, digit span forward and backward, structural MRI volumetric measurements, FLAIR (white matter hyperintensity, lacunes), CSF (Aβ_42_, p-tau, t-tau), FAQ, NPI, GDS, age, sex, education
	**Statistical models or algorithms:** DSI, CNN with Catboost, RF
	**Model performance:** AUC = 0.808–0.9, Accuracy = 0.766–0.87, Sensitivity = 0.736–0.968, Specificity = 0.189–0.86
SCD progression to MCI or AD dementia^48–50^	**Predictors:** MMSE, TMT-A, TMT-B, category fluency, MRI volumetric measurements, CSF (Aβ_42_, p-tau, t-tau), blood p-tau181, *APOE* genotype, sex, education
	**Statistical models or algorithms:** Cox regression, DSI, LR
	**Model performance:** AUC = 0.81–0.92, Accuracy = 0.741 (one study), Sensitivity = 0.757 (one study), Specificity = 0.726 (one study)
MCI progression to AD dementia^c,^^24,26,51–58^	**Predictors:** MMSE, CDR-SB, ADAS-Cog-11, ADAS-Cog-13, RAVLT, animal category fluency test, TMT-B, structural MRI volumetric measurements, FDG-PET features, CSF (Aβ_42_, p-tau, t-tau), FAQ, *APOE* genotype, comorbidities (e.g., diabetes), age, sex, education
	**Statistical models or algorithms:** Cox regression, Explainable Boosting Machine, LDA, LR, Naive Bayes, Polynomial Regression, SVM
	**Model performance:** AUC = 0.907–0.97, Accuracy = 0.81–0.917, Sensitivity = 0.793–0.967, Specificity = 0.77–0.898

AD: Alzheimer's disease; ADAS-Cog: Alzheimer's Disease Assessment Scale-cognitive subscale; AUC: Area under the Receiver Operating Characteristic Curve; BNT: Boston Naming Test; CDR-SB: Clinical Dementia Rating-Sum of Boxes; CNN: Convolutional Neural Network; CSF: cerebrospinal fluid; DSI: Disease State Index; ELM: Extreme Learning Machine; FAQ: Functional activities questionnaire; FDG-PET: fluorodeoxyglucose-positron emission tomography; FLAIR: Fluid attenuated inversion recovery; GBM: Gradient Boosting Machine; GDS: Geriatric Depression Scale; LDA: Linear discriminant analysis; LR: logistic regression; MCI: mild cognitive impairment; MMSE: Mini-Mental State Examination; MRI: Magnetic resonance imaging; NPI: Neuropsychiatric Inventory; RAVLT: Rey Auditory Verbal Learning Test; RF: Random Forest; SCD: subjective cognitive decline; SVM: Support Vector Machines; TMT: Trail Making Test.

Notes: ^a^All studies were based on ADNI. ^b^Three studies were based on ADNI. ^c^Seven studies were based on ADNI.

Despite having different predictive objectives, these models shared common sets of predictors, including global cognitive tests, cognitive domain-specific tests, MRI volumetric measurements, CSF biomarkers for AD, *APOE* genotype, and demographic information. Key differences included the incorporation of white matter hyperintensities and lacunes in models aimed at differentiating AD dementia from other types of dementia and FDG-PET in models predicting the progression of MCI to AD dementia. Since no systematic quality assessment was performed on the studies included in this literature review, the information should not be interpreted as representing the best prediction models for decision-making support. Instead, it can serve as a reference for future model development.

## Discussion

This literature review summarized more than a hundred multimodal prediction models for AD, more than half of which focused on predicting conversion from MCI to AD dementia. The multimodal prediction models performed well, with the majority of the models having an AUC above 0.8 and accuracy above 70%. However, more than 60% of the multimodal prediction models were developed using data from ADNI, and only around 10% of the studies conducted external validation for the prediction models.

Apart from predicting MCI progression to AD dementia, common objectives of existing multimodal prediction models included classifying the stages of AD (SCD versus MCI versus AD dementia), detecting the presence of amyloid, tau, or neurodegeneration, and differentiating AD dementia from other types of dementia. Relatively few studies aimed to predict the progression of SCD, even though it is a clinically important health outcome. Overall, the prediction models were consistent with the dementia workup and included cognitive tests, important AD neuroimaging (e.g., medial temporal atrophy observed on magnetic resonance imaging), CSF, and genetic predictors, and medical histories.^59^ The majority of the multimodal prediction models were based on machine learning algorithms ranging from classical machine learning algorithms to deep learning algorithms. This is in line with the high-dimensional nature of the predictors that were included in the prediction models. Nevertheless, despite their powerful predictive abilities, advanced algorithms are usually difficult to interpret, which may become a challenge to their application. Moreover, incorporating elements closely related to the outcomes of interest as main predictors and training models on small datasets may have inflated model performance. Our narrative synthesis suggests that this may have been the case for nearly half of the prediction models.

Existing prediction models have reported good performance. However, this literature review found that more than 60% of the prediction models were developed based on the ADNI dataset and lacked external validation. Black and Latinx individuals, as well as those with lower levels of education, are underrepresented in the ADNI dataset, suggesting that findings from existing literature may not generalize to populations outside of ADNI participants.^60^ A study found that associations between risk factors, cognitive function, and neuroimaging outcomes differed significantly in ADNI compared with a community-based study, which further emphasizes the importance of external validation.^61^ Another limitation of existing literature resulting from reliance on the ADNI dataset is that the diagnosis of MCI in the ADNI dataset requires subjective memory complaint and objective memory deficits (amnestic MCI). This constrains the ability to evaluate the predictive value of different MCI subtypes, such as non-amnestic and amnestic MCI. Additionally, consistent with a previous literature review of prediction models for MCI progression to AD dementia, we found that the performance of prediction models varied over different prediction horizons, even more within the same studies than across the studies.^12^ Therefore, it is important for the future development of prognostic models to evaluate the models over different prediction horizons and consider the clinical implications of different prediction horizons.

The findings of this literature review highlight the need for further research in several areas. Notably, there is an insufficient number of multimodal prediction models designed to predict the conversion of SCD to MCI or AD dementia, despite the significance of this outcome in AD progression. Existing prediction models should also be validated using data from more diverse populations and from both clinical and community-based settings. Additionally, no studies were identified that focused on developing multimodal prediction models for other critical health outcomes in individuals with AD, such as the risk of institutionalization or functional decline. Furthermore, novel blood-based biomarkers, such as p-tau217, have shown good performance in identifying AD pathology and can be considered for incorporation into future multimodal prediction models.^62^ Lastly, the increasing use of multiple high-dimensional data modalities combined with deep learning methods presents challenges for future evidence synthesis of AD prediction models. Although tools exist for reviewing the literature on prediction models, we found them impractical for multimodal AD prediction models due to the vast number of potential data modality combinations and data processing procedures.^63^ This highlights the need to develop specific tools for evidence syntheses of prognostic model studies in AD, a rapidly growing research area.

### Strengths and limitations

The major strength of this literature review is that its systematic literature search captured a large amount of literature on multimodal prediction models with various predictive objectives. This is important for giving an overview of the prediction models that have been developed and the need for further development of models with other predictive objectives. In addition, we conducted the review following the Arksey and O’Malley framework and adhered to the PRISMA-ScR checklist for reporting.

Several limitations of this literature review should be acknowledged. Firstly, the studies included in the literature review did not go through quality assessments. However, this literature review is a scoping review, for which qualitative synthesis is more emphasized while quality assessments are not required. In addition, our goal was to map the general performance and the main components of potentially useful multimodal prediction models rather than evaluate the potential biases in the models. Secondly, the studies included in the literature review, even when sharing the same predictive objective, had varying quality in terms of reporting their key information, such as the predictors included in the models and the performance of their models. Therefore, we could not extract detailed information from some studies with low reporting quality. Thirdly, our search missed some studies that did not include clear terminology that indicated the multiple data modalities in their models. Nonetheless, this literature review captured a large number of important predictors and statistical models or machine learning algorithms for multimodal prediction models for AD.

## Conclusions

This literature review suggests that many multimodal prediction models exist to predict relevant health outcomes in AD. These prediction models were mainly based on machine learning algorithms and included key aspects of the dementia workup. However, more than half of these models were trained with data from ADNI and need further validation before being applied to clinical practice.

## Supplemental Material

sj-docx-1-alz-10.1177_13872877251351630 - Supplemental material for Diagnostic and prognostic multimodal prediction models in Alzheimer's disease: A scoping review Supplemental material, sj-docx-1-alz-10.1177_13872877251351630 for Diagnostic and prognostic multimodal prediction models in Alzheimer's disease: A scoping review by Xin Xia, Lukas A Duffner, Christophe Bintener, Angela Bradshaw, Daphné Lamirel and Linus Jönsson in Journal of Alzheimer's Disease

sj-docx-2-alz-10.1177_13872877251351630 - Supplemental material for Diagnostic and prognostic multimodal prediction models in Alzheimer's disease: A scoping review Supplemental material, sj-docx-2-alz-10.1177_13872877251351630 for Diagnostic and prognostic multimodal prediction models in Alzheimer's disease: A scoping review by Xin Xia, Lukas A Duffner, Christophe Bintener, Angela Bradshaw, Daphné Lamirel and Linus Jönsson in Journal of Alzheimer's Disease

sj-docx-3-alz-10.1177_13872877251351630 - Supplemental material for Diagnostic and prognostic multimodal prediction models in Alzheimer's disease: A scoping review Supplemental material, sj-docx-3-alz-10.1177_13872877251351630 for Diagnostic and prognostic multimodal prediction models in Alzheimer's disease: A scoping review by Xin Xia, Lukas A Duffner, Christophe Bintener, Angela Bradshaw, Daphné Lamirel and Linus Jönsson in Journal of Alzheimer's Disease

sj-docx-4-alz-10.1177_13872877251351630 - Supplemental material for Diagnostic and prognostic multimodal prediction models in Alzheimer's disease: A scoping review Supplemental material, sj-docx-4-alz-10.1177_13872877251351630 for Diagnostic and prognostic multimodal prediction models in Alzheimer's disease: A scoping review by Xin Xia, Lukas A Duffner, Christophe Bintener, Angela Bradshaw, Daphné Lamirel and Linus Jönsson in Journal of Alzheimer's Disease

sj-docx-5-alz-10.1177_13872877251351630 - Supplemental material for Diagnostic and prognostic multimodal prediction models in Alzheimer's disease: A scoping review Supplemental material, sj-docx-5-alz-10.1177_13872877251351630 for Diagnostic and prognostic multimodal prediction models in Alzheimer's disease: A scoping review by Xin Xia, Lukas A Duffner, Christophe Bintener, Angela Bradshaw, Daphné Lamirel and Linus Jönsson in Journal of Alzheimer's Disease
