# Supplementary material for: Diagnostic and prognostic multimodal prediction models in Alzheimer's disease: A scoping review
Source: J Alzheimers Dis. 2025 Jun 26;108(1 Suppl):S209–21. doi: 10.1177/13872877251351630 (PMC12583647; doi:10.1177/13872877251351630)
Supplement: sj-docx-2-alz-10.1177_13872877251351630 - Supplemental material for Diagnostic and prognostic multimodal prediction models in Alzheimer's disease: A scoping review [file sj-docx-2-alz-10.1177_13872877251351630.docx]

## **Search strategy in Medline**

| **#** | **Searches** | **Results** |
| --- | --- | --- |
| 1 | Dementia/ | 63049 |
| 2 | Alzheimer Disease/ | 122432 |
| 3 | Cognitive Dysfunction/ | 38181 |
| 4 | (alzheimer* or dementia*).ti,ab,kf. | 287227 |
| 5 | (cogniti* adj3 (decline or defect* or deficit* or disabilit* or disorder* or dysfunction* or impairment*)).ti,ab,kf. | 178426 |
| 6 | or/1-5 | 420797 |
| 7 | Disease Progression/ | 191578 |
| 8 | Early Diagnosis/ | 30498 |
| 9 | diagnosis.fs. | 2986995 |
| 10 | (assess* or predict* or probabilistic classification or risk identification*).ti,ab,kf. | 5538619 |
| 11 | (disease adj3 (exacerbation or process or progress*)).ti,ab,kf. | 226379 |
| 12 | (early detect* or early diagnos* or early identification or discriminat*).ti,ab,kf. | 515686 |
| 13 | ((detect* or diagnos*) adj3 (alzheimer* or AD)).ti,ab,kf. | 16165 |
| 14 | ((convert* or prognos*) and ((mild cognitive impairment* or MCI or subjective cognitive decline) and (alzheimer* or AD or dementia*))).ti,ab,kf. | 2007 |
| 15 | or/7-14 | 8351157 |
| 16 | exp Neural Networks, Computer/ | 63793 |
| 17 | Machine Learning/ | 36492 |
| 18 | (deep learning or machine learning or multilayer* or multi-layer* or multimodal* or multi-modal* or multitask* learning or multi-task* learning).ti,ab,kf. | 268787 |
| 19 | ((multidimensional or multi-dimensional or multidomain* or multi-domain* or multifactor* or multi-factor*) adj4 (assessment* or data* or evaluat* or instrument* or measure* or model* or predict* or risk model* or risk predict* or tool*)).ti,ab,kf. | 17396 |
| 20 | (algorithmic neural network* or artificial neural network* or comput* neural network* or connectionist model* or neural network model* or perceptron*).ti,ab,kf. | 29323 |
| 21 | or/16-20 | 331312 |
| 22 | 6 and 15 and 21 | 5755 |
| 23 | limit 22 to english | 5630 |

## **Search strategy in Embase**

| **No.** | **Query** | **Results** |
| --- | --- | --- |
| #26 | #8 AND #17 AND #24 AND [english]/lim | 8496 |
| #25 | #8 AND #17 AND #24 | 8638 |
| #24 | #18 OR #19 OR #20 OR #21 OR #22 OR #23 | 409752 |
| #23 | 'algorithmic neural network*':ti,ab,kw OR 'artificial neural network*':ti,ab,kw OR 'comput* neural network*':ti,ab,kw OR 'connectionist model*':ti,ab,kw OR 'neural network model*':ti,ab,kw OR perceptron*:ti,ab,kw | 33909 |
| #22 | ((multidimensional OR 'multi dimensional' OR multidomain* OR 'multi domain*' OR multifactor* OR 'multi factor*') NEAR/4 (assessment* OR data* OR evaluat* OR instrument* OR measure* OR model* OR predict* OR 'risk model*' OR 'risk predict*' OR tool*)):ti,ab,kw | 22113 |
| #21 | 'deep learning':ti,ab,kw OR 'machine learning':ti,ab,kw OR multilayer*:ti,ab,kw OR 'multi layer*':ti,ab,kw OR multimodal*:ti,ab,kw OR 'multi modal*':ti,ab,kw OR 'multitask* learning':ti,ab,kw OR 'multi-task* learning':ti,ab,kw | 322105 |
| #20 | 'machine learning'/de | 107758 |
| #19 | 'deep learning'/de | 50257 |
| #18 | 'artificial neural network'/de | 54497 |
| #17 | #9 OR #10 OR #11 OR #12 OR #13 OR #14 OR #15 OR #16 | 8479145 |
| #16 | (convert*:ti,ab,kw OR prognos*:ti,ab,kw) AND ('mild cognitive impairment*':ti,ab,kw OR mci:ti,ab,kw OR 'subjective cognitive decline':ti,ab,kw) AND (alzheimer*:ti,ab,kw OR ad:ti,ab,kw OR dementia*:ti,ab,kw) | 3536 |
| #15 | ((detect* OR diagnos*) NEAR/3 (alzheimer* OR ad)):ti,ab,kw | 24774 |
| #14 | 'early detect*':ti,ab,kw OR 'early diagnos*':ti,ab,kw OR 'early identification':ti,ab,kw OR discriminat*:ti,ab,kw | 684490 |
| #13 | (disease NEAR/3 (exacerbation OR process OR progress*)):ti,ab,kw | 375438 |
| #12 | assess*:ti,ab,kw OR predict*:ti,ab,kw OR 'probabilistic classification':ti,ab,kw OR 'risk identification*':ti,ab,kw | 7650358 |
| #11 | 'dementia assessment'/de OR 'alzheimer disease assessment scale'/de | 3661 |
| #10 | 'early diagnosis'/de | 131890 |
| #9 | 'disease exacerbation'/de | 193892 |
| #8 | #1 OR #2 OR #3 OR #4 OR #5 OR #6 OR #7 | 689370 |
| #7 | (cogniti* NEAR/3 (decline OR defect* OR deficit* OR disabilit* OR disorder* OR dysfunction* OR impairment*)):ti,ab,kw | 266168 |
| #6 | alzheimer*:ti,ab,kw OR dementia*:ti,ab,kw | 397689 |
| #5 | 'cognitive decline'/de | 193 |
| #4 | 'mild cognitive impairment'/de | 38968 |
| #3 | 'cognitive defect'/de | 224068 |
| #2 | 'alzheimer disease'/de | 254317 |

## **Search strategy in Web of Science**

| **#** | **Search Query** | **Results** |
| --- | --- | --- |
| 1 | TS=(cogniti* NEAR/3 (decline OR defect* OR deficit* OR disabilit* OR disorder* OR dysfunction* OR impairment*)) | 230126 |
| 2 | TS=(alzheimer* OR dementia*) | 402929 |
| 3 | #1 OR #2 | 529162 |
| 4 | TS=(assess* OR predict* OR "probabilistic classification" OR "risk identification*") | 8411201 |
| 5 | TS=(disease NEAR/3 (exacerbation OR process OR progress*)) | 233623 |
| 6 | TS=("early detect*" OR "early diagnos*" OR "early identification" OR discriminat*) | 696960 |
| 7 | TS=((detect* OR diagnos*) NEAR/3 (alzheimer* OR AD)) | 19146 |
| 8 | TS=(convert* OR prognos*) | 1491836 |
| 9 | #4 OR #5 OR #6 OR #7 OR #8 | 10038251 |
| 10 | TS=("deep learning" OR "machine learning" OR multilayer* OR multi-layer* OR multimodal* OR multi-modal* OR "multitask* learning" OR "multi-task* learning") | 697089 |
| 11 | TS=((multidimensional OR multi-dimensional OR multidomain* OR multi-domain* OR multifactor* OR multi-factor*) NEAR/4 (assessment* OR data* OR evaluat* OR instrument* OR measure* OR model* OR predict* OR "risk model*" OR "risk predict*" OR tool*)) | 38810 |
| 12 | TS=("algorithmic neural network*" OR "artificial neural network*" OR "comput* neural network*" OR "connectionist model*" OR "neural network model*" OR perceptron*) | 134786 |
| 13 | #10 OR #11 OR #12 | 822070 |
| 14 | #3 AND #9 AND #13 | 6705 |
| 15 | #3 AND #9 AND #13 and English (Languages) | 6614 |
