# Supplementary material for: Diagnostic and prognostic multimodal prediction models in Alzheimer's disease: A scoping review
Source: J Alzheimers Dis. 2025 Jun 26;108(1 Suppl):S209–21. doi: 10.1177/13872877251351630 (PMC12583647; doi:10.1177/13872877251351630)
Supplement: sj-docx-3-alz-10.1177_13872877251351630 - Supplemental material for Diagnostic and prognostic multimodal prediction models in Alzheimer's disease: A scoping review [file sj-docx-3-alz-10.1177_13872877251351630.docx]

1. Apostolova LG, Hwang KS, Avila D, Elashoff D, Kohannim O, Teng E, et al. Brain amyloidosis ascertainment from cognitive, imaging, and peripheral blood protein measures. Neurology. 2015;84(7):729-37.

2. Apostolova LG, Hwang KS, Kohannim O, Avila D, Elashoff D, Jack CR, et al. ApoE4 effects on automated diagnostic classifiers for mild cognitive impairment and Alzheimer's disease. Neuroimage Clin. 2014;4:461-72.

3. Arco JE, Ramírez J, Górriz JM, Ruz M. Data fusion based on Searchlight analysis for the prediction of Alzheimer’s disease. Expert Syst Appl. 2021;185:115549.

4. Ardekani BA, Bermudez E, Mubeen AM, Bachman AH. Prediction of Incipient Alzheimer's Disease Dementia in Patients with Mild Cognitive Impairment. J Alzheimers Dis. 2017;55(1):269-81.

5. Avelar-Pereira B, Belloy ME, O’Hara R, Hosseini SMH, for the Alzheimer’s Disease Neuroimaging I. Decoding the heterogeneity of Alzheimer’s disease diagnosis and progression using multilayer networks. Mol Psychiatry. 2023;28(6):2423-32.

6. Bapat R, Ma D, Duong TQ. Predicting Four-Year's Alzheimer's Disease Onset Using Longitudinal Neurocognitive Tests and MRI Data Using Explainable Deep Convolutional Neural Networks. J Alzheimers Dis. 2024;97(1):459-69.

7. Bauer CM, Cabral HJ, Killiany RJ. Multimodal Discrimination between Normal Aging, Mild Cognitive Impairment and Alzheimer's Disease and Prediction of Cognitive Decline. Diagnostics. 2018;8(1).

8. Bruun M, Rhodius-Meester HFM, Koikkalainen J, Baroni M, Gjerum L, Lemstra AW, et al. Evaluating combinations of diagnostic tests to discriminate different dementia types. Alzheimers Dement. 2018;10:509-18.

9. Bucholc M, Titarenko S, Ding X, Canavan C, Chen T. A hybrid machine learning approach for prediction of conversion from mild cognitive impairment to dementia. Expert Syst Appl. 2023;217:119541.

10. Cai J, Hu W, Ma J, Si A, Chen S, Gong L, et al. Explainable Machine Learning with Pairwise Interactions for Predicting Conversion from Mild Cognitive Impairment to Alzheimer’s Disease Utilizing Multi-Modalities Data. Brain Sci. 2023;13(11):1535.

11. Cai Y, Fan X, Zhao L, Liu W, Luo Y, Lau AYL, et al. Comparing machine learning-derived MRI-based and blood-based neurodegeneration biomarkers in predicting syndromal conversion in early AD. Alzheimers Dement. 2023;19(11):4987-98.

12. Cao E, Ma D, Nayak S, Duong TQ. Deep learning combining FDG-PET and neurocognitive data accurately predicts MCI conversion to Alzheimer's dementia 3-year post MCI diagnosis. Neurobiol Dis. 2023;187:106310.

13. Chang M, Brainerd CJ. Predicting conversion from mild cognitive impairment to Alzheimer's disease with multimodal latent factors. J Clin Exp Neuropsychol. 2022;44(4):316-35.

14. Chen H, Guo H, Xing L, Chen D, Yuan T, Zhang Y, Zhang X. Multimodal predictive classification of Alzheimer's disease based on attention-combined fusion network: Integrated neuroimaging modalities and medical examination data. IET Image Process. 2023;17(11):3153-64.

15. Chen J, Chen G, Shu H, Chen G, Ward BD, Wang Z, et al. Predicting progression from mild cognitive impairment to Alzheimer's disease on an individual subject basis by applying the CARE index across different independent cohorts. Aging. 2019;11(8):2185-201.

16. Cheng B, Liu M, Zhang D, Munsell B, Shen D. Domain Transfer Learning for MCI Conversion Prediction. IEEE Trans Biomed Eng. 2015;62(7):1805-17.

17. Cheng B, Zhang D, Chen S, Kaufer DI, Shen D. Semi-supervised multimodal relevance vector regression improves cognitive performance estimation from imaging and biological biomarkers. Neuroinformatics. 2013;11(3):339-53.

18. Cheng B, Zhu B, Pu S. Multi-auxiliary domain transfer learning for diagnosis of MCI conversion. Neurol Sci. 2022;43(3):1721-39.

19. Cheng X, Li D, Peng J, Shu Z, Xing X. Prediction of Mild Cognitive Impairment Progression to Alzheimer's Disease Based on Diffusion Tensor Imaging-Derived Diffusion Parameters: Construction and Validation of a Nomogram. Eur Neurol. 2023;86(6):408-17.

20. Chiu SI, Fan LY, Lin CH, Chen TF, Lim WS, Jang JR, Chiu MJ. Machine Learning-Based Classification of Subjective Cognitive Decline, Mild Cognitive Impairment, and Alzheimer’s Dementia Using Neuroimage and Plasma Biomarkers. ACS Chem Neurosci. 2022;13(23):3263-70.

21. Chun MY, Park CJ, Kim J, Jeong JH, Jang H, Kim K, Seo SW. Prediction of conversion to dementia using interpretable machine learning in patients with amnestic mild cognitive impairment. Front Aging Neurosci. 2022;14:898940.

22. Cui Y, Liu B, Luo S, Zhen X, Fan M, Liu T, et al. Identification of conversion from mild cognitive impairment to Alzheimer's disease using multivariate predictors. PLoS One. 2011;6(7):e21896.

23. Dauwan M, van der Zande JJ, van Dellen E, Sommer IE, Scheltens P, Lemstra AW, Stam CJ. Random forest to differentiate dementia with Lewy bodies from Alzheimer's disease. Alzheimers Dement. 2016;4:99-106.

24. Devanand DP, Liu X, Brown PJ, Huey ED, Stern Y, Pelton GH. A two-study comparison of clinical and MRI markers of transition from mild cognitive impairment to Alzheimer's disease. Int J Alzheimers Dis. 2012;2012:483469.

25. Devanand DP, Liu X, Tabert MH, Pradhaban G, Cuasay K, Bell K, et al. Combining early markers strongly predicts conversion from mild cognitive impairment to Alzheimer's disease. Biol Psychiatry. 2008;64(10):871-9.

26. Dolcet-Negre MM, Imaz Aguayo L, García-de-Eulate R, Martí-Andrés G, Fernández-Matarrubia M, Domínguez P, et al. Predicting Conversion from Subjective Cognitive Decline to Mild Cognitive Impairment and Alzheimer's Disease Dementia Using Ensemble Machine Learning. J Alzheimers Dis. 2023;93(1):125-40.

27. Dukart. J, Sambataro. F, Bertolino. A. Accurate Prediction of Conversion to Alzheimer's Disease using Imaging, Genetic, and Neuropsychological Biomarkers. J Alzheimers Dis. 2016;49(4):1143-59.

28. El-Sappagh S, Alonso JM, Islam SMR, Sultan AM, Kwak KS. A multilayer multimodal detection and prediction model based on explainable artificial intelligence for Alzheimer's disease. Sci Rep. 2021;11(1):2660.

29. El-Sappagh S, Saleh H, Ali F, Amer E, Abuhmed T. Two-stage deep learning model for Alzheimer’s disease detection and prediction of the mild cognitive impairment time. Neural Comput Appl. 2022;34(17):14487-509.

30. El-Sappagh S, Saleh H, Sahal R, Abuhmed T, Islam SMR, Ali F, Amer E. Alzheimer’s disease progression detection model based on an early fusion of cost-effective multimodal data. Future Gener Comput Syst. 2021;115:680-99.

31. Escudero J, Ifeachor E, Zajicek JP, Green C, Shearer J, Pearson S. Machine Learning-Based Method for Personalized and Cost-Effective Detection of Alzheimer's Disease. IEEE Trans Biomed Eng. 2013;60(1):164-8.

32. Ewers M, Walsh C, Trojanowski JQ, Shaw LM, Petersen RC, Jack CR, Jr., et al. Prediction of conversion from mild cognitive impairment to Alzheimer's disease dementia based upon biomarkers and neuropsychological test performance. Neurobiol Aging. 2012;33(7):1203-14.

33. Ezzati A, Abdulkadir A, Jack CR, Jr., Thompson PM, Harvey DJ, Truelove-Hill M, et al. Predictive value of ATN biomarker profiles in estimating disease progression in Alzheimer's disease dementia. Alzheimers Dement. 2021;17(11):1855-67.

34. Ezzati A, Davatzikos C, Wolk DA, Hall CB, Habeck C, Lipton RB. Application of predictive models in boosting power of Alzheimer's disease clinical trials: A post hoc analysis of phase 3 solanezumab trials. Alzheimers Dement. 2022;8(1):e12223.

35. Ezzati A, Harvey DJ, Habeck C, Golzar A, Qureshi IA, Zammit AR, et al. Predicting Amyloid-β Levels in Amnestic Mild Cognitive Impairment Using Machine Learning Techniques. J Alzheimers Dis. 2020;73(3):1211-9.

36. Ezzati A, Zammit AR, Harvey DJ, Habeck C, Hall CB, Lipton RB. Optimizing Machine Learning Methods to Improve Predictive Models of Alzheimer's Disease. J Alzheimers Dis. 2019;71(3):1027-36.

37. Franciotti R, Nardini D, Russo M, Onofrj M, Sensi SL. Comparison of Machine Learning-based Approaches to Predict the Conversion to Alzheimer's Disease from Mild Cognitive Impairment. Neuroscience. 2023;514:143-52.

38. Franzmeier N, Koutsouleris N, Benzinger T, Goate A, Karch CM, Fagan AM, et al. Predicting sporadic Alzheimer's disease progression via inherited Alzheimer's disease-informed machine-learning. Alzheimers Dement. 2020;16(3):501-11.

39. Frölich L, Peters O, Lewczuk P, Gruber O, Teipel SJ, Gertz HJ, et al. Incremental value of biomarker combinations to predict progression of mild cognitive impairment to Alzheimer's dementia. Alzheimers Res Ther. 2017;9(1):84.

40. Gao F, Lv X, Dai L, Wang Q, Wang P, Cheng Z, et al. A combination model of AD biomarkers revealed by machine learning precisely predicts Alzheimer's dementia: China Aging and Neurodegenerative Initiative (CANDI) study. Alzheimers Dement. 2023;19(3):749-60.

41. Ghafoori S, Shalbaf A. Predicting conversion from MCI to AD by integration of rs-fMRI and clinical information using 3D-convolutional neural network. Int J Comput Assist Radiol Surg. 2022;17(7):1245-55.

42. Gjerum L, Frederiksen KS, Henriksen OM, Law I, Bruun M, Simonsen AH, et al. Evaluating 2-[(18)F]FDG-PET in differential diagnosis of dementia using a data-driven decision model. Neuroimage Clin. 2020;27:102267.

43. Goryawala M, Zhou Q, Barker W, Loewenstein DA, Duara R, Adjouadi M. Inclusion of Neuropsychological Scores in Atrophy Models Improves Diagnostic Classification of Alzheimer's Disease and Mild Cognitive Impairment. Comput Intell Neurosci. 2015;2015:865265.

44. Grassi M, Loewenstein DA, Caldirola D, Schruers K, Duara R, Perna G. A clinically-translatable machine learning algorithm for the prediction of Alzheimer’s disease conversion: further evidence of its accuracy via a transfer learning approach. Int Psychogeriatr. 2019;31(7):937-45.

45. Grassi M, Rouleaux N, Caldirola D, Loewenstein D, Schruers K, Perna G, Dumontier M. A Novel Ensemble-Based Machine Learning Algorithm to Predict the Conversion From Mild Cognitive Impairment to Alzheimer's Disease Using Socio-Demographic Characteristics, Clinical Information, and Neuropsychological Measures. Front Neurol. 2019;10:756.

46. Gupta Y, Kim JI, Kim BC, Kwon GR. Classification and Graphical Analysis of Alzheimer's Disease and Its Prodromal Stage Using Multimodal Features From Structural, Diffusion, and Functional Neuroimaging Data and the APOE Genotype. Front Aging Neurosci. 2020;12:238.

47. Gupta Y, Lama RK, Kwon GR. Prediction and Classification of Alzheimer's Disease Based on Combined Features From Apolipoprotein-E Genotype, Cerebrospinal Fluid, MR, and FDG-PET Imaging Biomarkers. Front Comput Neurosci. 2019;13:72.

48. Hall A, Muñoz-Ruiz M, Mattila J, Koikkalainen J, Tsolaki M, Mecocci P, et al. Generalizability of the disease state index prediction model for identifying patients progressing from mild cognitive impairment to Alzheimer's disease. J Alzheimers Dis. 2015;44(1):79-92.

49. Ho NH, Jeong YH, Kim J. Multimodal multitask learning for predicting MCI to AD conversion using stacked polynomial attention network and adaptive exponential decay. Sci Rep. 2023;13(1):11243.

50. Huang K, Lin Y, Yang L, Wang Y, Cai S, Pang L, et al. A multipredictor model to predict the conversion of mild cognitive impairment to Alzheimer's disease by using a predictive nomogram. Neuropsychopharmacology. 2020;45(2):358-66.

51. Hwang J, Park HK, Yoon HJ, Jeong JH, Lee H. Detecting amyloid-β positivity using regions of interest from structural magnetic resonance imaging. Eur J Neurol. 2023;30(6):1574-84.

52. Iddi S, Li D, Aisen PS, Rafii MS, Thompson WK, Donohue MC. Predicting the course of Alzheimer's progression. Brain Inform. 2019;6(1):6.

53. Jang H, Park J, Woo S, Kim S, Kim HJ, Na DL, et al. Prediction of fast decline in amyloid positive mild cognitive impairment patients using multimodal biomarkers. Neuroimage Clin. 2019;24:101941.

54. Jie B, Zhang D, Cheng B, Shen D. Manifold regularized multitask feature learning for multimodality disease classification. Hum Brain Mapp. 2015;36(2):489-507.

55. Jo S, Lee H, Kim HJ, Suh CH, Kim SJ, Lee Y, et al. Do radiomics or diffusion-tensor images provide additional information to predict brain amyloid-beta positivity? Sci Rep. 2023;13(1):9755.

56. Joo SH, Lee CU. Cerebral Amyloid Positivity Prediction Models Using Clinical Data in Subjects With Mild Cognitive Impairment and Dementia. Psychiatry Investig. 2021;18(9):864-70.

57. K. P MN, P T. Alzheimer's classification using dynamic ensemble of classifiers selection algorithms: A performance analysis. Biomed Signal Process Control. 2021;68:102729.

58. Kang SH, Cheon BK, Kim JS, Jang H, Kim HJ, Park KW, et al. Machine learning for the prediction of amyloid positivity in amnestic mild cognitive impairment. J Alzheimers Dis. 2021;80(1):143-57.

59. Khatri U, Kwon GR. An Efficient Combination among sMRI, CSF, Cognitive Score, and APOE ε 4 Biomarkers for Classification of AD and MCI Using Extreme Learning Machine. Comput Intell Neurosci. 2020;2020:8015156.

60. Kikuchi M, Kobayashi K, Itoh S, Kasuga K, Miyashita A, Ikeuchi T, et al. Identification of mild cognitive impairment subtypes predicting conversion to Alzheimer’s disease using multimodal data. Comput Struct Biotechnol J. 2022;20:5296-308.

61. Kim J, Park Y, Park S, Jang H, Kim HJ, Na DL, et al. Prediction of tau accumulation in prodromal Alzheimer's disease using an ensemble machine learning approach. Sci Rep. 2021;11(1):5706.

62. Kim JP, Kim J, Jang H, Kim J, Kang SH, Kim JS, et al. Predicting amyloid positivity in patients with mild cognitive impairment using a radiomics approach. Sci Rep. 2021;11(1):6954.

63. Kim SE, Woo S, Kim SW, Chin J, Kim HJ, Lee BI, et al. A Nomogram for Predicting Amyloid PET Positivity in Amnestic Mild Cognitive Impairment. J Alzheimers Dis. 2018;66(2):681-91.

64. Kivisäkk P, Magdamo C, Trombetta BA, Noori A, Kuo YKE, Chibnik LB, et al. Plasma biomarkers for prognosis of cognitive decline in patients with mild cognitive impairment. Brain Commun. 2022;4(4):fcac155.

65. Ko H, Park S, Kwak S, Ihm J, for the ARG. Exploring a Cost-Efficient Model for Predicting Cerebral Aβ Burden Using MRI and Neuropsychological Markers in the ADNI-2 Cohort. J Pers Med. 2020;10(4):197.

66. Korolev IO, Symonds LL, Bozoki AC. Predicting Progression from Mild Cognitive Impairment to Alzheimer's Dementia Using Clinical, MRI, and Plasma Biomarkers via Probabilistic Pattern Classification. PLoS One. 2016;11(2):e0138866.

67. Kuang J, Zhang P, Cai T, Zou Z, Li L, Wang N, Wu L. Prediction of transition from mild cognitive impairment to Alzheimer's disease based on a logistic regression-artificial neural network-decision tree model. Geriatr Gerontol Int. 2021;21(1):43-7.

68. Lee G, Nho K, Kang B, Sohn KA, Kim D. Predicting Alzheimer's disease progression using multi-modal deep learning approach. Sci Rep. 2019;9(1):1952.

69. Lehallier B, Essioux L, Gayan J, Alexandridis R, Nikolcheva T, Wyss-Coray T, Britschgi M. Combined Plasma and Cerebrospinal Fluid Signature for the Prediction of Midterm Progression From Mild Cognitive Impairment to Alzheimer Disease. JAMA Neurol. 2016;73(2):203-12.

70. Li S, Okonkwo O, Albert M, Wang MC. Variation in Variables that Predict Progression from MCI to AD Dementia over Duration of Follow-up. Am J Alzheimers Dis. 2013;2(1):12-28.

71. Lin L, Xiong M, Zhang G, Kang W, Sun S, Wu S. A Convolutional Neural Network and Graph Convolutional Network Based Framework for AD Classification. Sensors. 2023;23(4).

72. Lin W, Gao Q, Yuan J, Chen Z, Feng C, Chen W, et al. Predicting Alzheimer's Disease Conversion From Mild Cognitive Impairment Using an Extreme Learning Machine-Based Grading Method With Multimodal Data. Front Aging Neurosci. 2020;12:77.

73. Liu L, Liu S, Zhang L, To XV, Nasrallah F, Chandra SS. Cascaded Multi-Modal Mixing Transformers for Alzheimer’s Disease Classification with Incomplete Data. NeuroImage. 2023;277:120267.

74. Liu Y, Mattila J, Ruiz M, Paajanen T, Koikkalainen J, van Gils M, et al. Predicting AD conversion: comparison between prodromal AD guidelines and computer assisted PredictAD tool. PLoS One. 2013;8(2):e55246.

75. Martinez-Torteya A, Rodriguez-Rojas J, Celaya-Padilla JM, Galván-Tejada JI, Treviño V, Tamez-Peña J. Magnetization-prepared rapid acquisition with gradient echo magnetic resonance imaging signal and texture features for the prediction of mild cognitive impairment to Alzheimer's disease progression. J Med Imaging. 2014;1(3):031005.

76. Martínez-Torteya A, Treviño V, Tamez-Peña JG. Improved Diagnostic Multimodal Biomarkers for Alzheimer’s Disease and Mild Cognitive Impairment. Biomed Res Int. 2015;2015:961314.

77. Massetti N, Russo M, Franciotti R, Nardini D, Mandolini GM, Granzotto A, et al. A Machine Learning-Based Holistic Approach to Predict the Clinical Course of Patients within the Alzheimer’s Disease Spectrum. J Alzheimers Dis. 2022;85:1639-55.

78. Mattila J, Soininen H, Koikkalainen J, Rueckert D, Wolz R, Waldemar G, Lötjönen J. Optimizing the diagnosis of early Alzheimer's disease in mild cognitive impairment subjects. J Alzheimers Dis. 2012;32(4):969-79.

79. Mattoli MV, Cocciolillo F, Chiacchiaretta P, Dotta F, Trevisi G, Carrarini C, et al. Combined 18F-FDG PET-CT markers in dementia with Lewy bodies. Alzheimers Dement. 2023;15(4):e12515.

80. McCombe N, Joshi A, Finn DP, McClean PL, Roberts G, O'Brien JT, et al. Distinguishing Lewy Body Dementia from Alzheimer's Disease using Machine Learning on Heterogeneous Data: A Feasibility Study. Annu Int Conf IEEE Eng Med Biol Soc. 2022;2022:4929-33.

81. Minhas S, Khanum A, Riaz F, Alvi A, Khan SA. A Nonparametric Approach for Mild Cognitive Impairment to AD Conversion Prediction: Results on Longitudinal Data. IEEE J Biomed Health Inform. 2017;21(5):1403-10.

82. Minhas S, Khanum A, Riaz F, Khan SA, Alvi A. Predicting Progression From Mild Cognitive Impairment to Alzheimer's Disease Using Autoregressive Modelling of Longitudinal and Multimodal Biomarkers. IEEE J Biomed Health Inform. 2018;22(3):818-25.

83. Mirabnahrazam G, Ma D, Beaulac C, Lee S, Popuri K, Lee H, et al. Predicting time-to-conversion for dementia of Alzheimer's type using multi-modal deep survival analysis. Neurobiol Aging. 2023;121:139-56.

84. Mofrad SA, Lundervold AJ, Vik A, Lundervold AS. Cognitive and MRI trajectories for prediction of Alzheimer’s disease. Sci Rep. 2021;11(1):2122.

85. Moradi E, Pepe A, Gaser C, Huttunen H, Tohka J. Machine learning framework for early MRI-based Alzheimer's conversion prediction in MCI subjects. Neuroimage. 2015;104:398-412.

86. Moreira LB, Namen AA. A hybrid data mining model for diagnosis of patients with clinical suspicion of dementia. Comput Methods Programs Biomed. 2018;165:139-49.

87. Nguyen K, Nguyen M, Dang K, Pham B, Huynh V, Vo T, et al. Early Alzheimer's disease diagnosis using an XG-Boost model applied to MRI images. Biomed Res Ther. 2023;10(9):5896-911.

88. Palmqvist S, Hertze J, Minthon L, Wattmo C, Zetterberg H, Blennow K, et al. Comparison of brief cognitive tests and CSF biomarkers in predicting Alzheimer's disease in mild cognitive impairment: six-year follow-up study. PLoS One. 2012;7(6):e38639.

89. Palmqvist S, Insel PS, Zetterberg H, Blennow K, Brix B, Stomrud E, et al. Accurate risk estimation of β-amyloid positivity to identify prodromal Alzheimer's disease: Cross-validation study of practical algorithms. Alzheimers Dement. 2019;15(2):194-204.

90. Palmqvist S, Tideman P, Cullen N, Zetterberg H, Blennow K, Dage JL, et al. Prediction of future Alzheimer's disease dementia using plasma phospho-tau combined with other accessible measures. Nat Med. 2021;27(6):1034-42.

91. Park CJ, Seo Y, Choe YS, Jang H, Lee H, Kim JP. Predicting conversion of brain β-amyloid positivity in amyloid-negative individuals. Alzheimers Res Ther. 2022;14(1):129.

92. Park HJ, Lee JY, Yang JJ, Kim HJ, Kim YS, Kim JY, Choi YY. Prediction of Amyloid β-Positivity with both MRI Parameters and Cognitive Function Using Machine Learning. J Korean Soc Radiol. 2023;84(3):638-52.

93. Pena D, Suescun J, Schiess M, Ellmore TM, Giancardo L. Toward a Multimodal Computer-Aided Diagnostic Tool for Alzheimer's Disease Conversion. Front Neurosci. 2021;15:744190.

94. Peters F, Villeneuve S, Belleville S. Predicting progression to dementia in elderly subjects with mild cognitive impairment using both cognitive and neuroimaging predictors. J Alzheimers Dis. 2014;38(2):307-18.

95. Planche V, Bouteloup V, Pellegrin I, Mangin JF, Dubois B, Ousset PJ, et al. Validity and Performance of Blood Biomarkers for Alzheimer Disease to Predict Dementia Risk in a Large Clinic-Based Cohort. Neurology. 2023;100(5):e473-e84.

96. Platero C, Tobar MC. Predicting Alzheimer's conversion in mild cognitive impairment patients using longitudinal neuroimaging and clinical markers. Brain Imaging Behav. 2021;15(4):1728-38.

97. Qiang YR, Zhang SW, Li JN, Li Y, Zhou QY. Diagnosis of Alzheimer’s disease by joining dual attention CNN and MLP based on structural MRIs, clinical and genetic data. Artif Intell Med. 2023;145:102678.

98. Qiu S, Miller MI, Joshi PS, Lee JC, Xue C, Ni Y, et al. Multimodal deep learning for Alzheimer's disease dementia assessment. Nat Commun. 2022;13(1):3404.

99. Reas ET, Shadrin A, Frei O, Motazedi E, McEvoy L, Bahrami S, et al. Improved multimodal prediction of progression from MCI to Alzheimer's disease combining genetics with quantitative brain MRI and cognitive measures. Alzheimers Dement. 2023;19(11):5151-8.

100. Rhodius-Meester HFM, Liedes H, Koikkalainen J, Wolfsgruber S, Coll-Padros N, Kornhuber J, et al. Computer-assisted prediction of clinical progression in the earliest stages of AD. Alzheimers Dement. 2018;10:726-36.

101. Ritter K, Schumacher J, Weygandt M, Buchert R, Allefeld C, Haynes JD. Multimodal prediction of conversion to Alzheimer's disease based on incomplete biomarkers. Alzheimers Dement. 2015;1(2):206-15.

102. Rye I, Vik A, Kocinski M, Lundervold AS, Lundervold AJ. Predicting conversion to Alzheimer's disease in individuals with Mild Cognitive Impairment using clinically transferable features. Sci Rep. 2022;12(1):15566.

103. Segovia F, Bastin C, Salmon E, Górriz JM, Ramírez J, Phillips C. Combining PET Images and Neuropsychological Test Data for Automatic Diagnosis of Alzheimer's Disease. PLoS One. 2014;9(2):e88687.

104. Shaffer JL, Petrella JR, Sheldon FC, Choudhury KR, Calhoun VD, Coleman RE, Doraiswamy PM. Predicting cognitive decline in subjects at risk for Alzheimer disease by using combined cerebrospinal fluid, MR imaging, and PET biomarkers. Radiology. 2013;266(2):583-91.

105. Shafiee N, Dadar M, Ducharme S, Collins DL, for the Alzheimer’s Disease Neuroimaging I. Automatic Prediction of Cognitive and Functional Decline Can Significantly Decrease the Number of Subjects Required for Clinical Trials in Early Alzheimer’s Disease. J Alzheimers Dis. 2021;84:1071-8.

106. Shu ZY, Mao DW, Xu YY, Shao Y, Pang PP, Gong XY. Prediction of the progression from mild cognitive impairment to Alzheimer's disease using a radiomics-integrated model. Ther Adv Neurol Disord. 2021;14:17562864211029551.

107. Simonsen AH, Mattila J, Hejl AM, Garde E, van Gils M, Thomsen C, et al. Application of the PredictAD decision support tool to a Danish cohort of patients with Alzheimer's disease and other dementias. Dement Geriatr Cogn Disord. 2014;37(3-4):207-13.

108. Spasov S, Passamonti L, Duggento A, Liò P, Toschi N. A parameter-efficient deep learning approach to predict conversion from mild cognitive impairment to Alzheimer's disease. Neuroimage. 2019;189:276-87.

109. Sørensen L, Nielsen M. Ensemble support vector machine classification of dementia using structural MRI and mini-mental state examination. J Neurosci Methods. 2018;302:66-74.

110. Tam A, Dansereau C, Iturria-Medina Y, Urchs S, Orban P, Sharmarke H, et al. A highly predictive signature of cognition and brain atrophy for progression to Alzheimer's dementia. GigaScience. 2019;8(5):giz055.

111. Tam A, Laurent C, Gauthier S, Dansereau C. Prediction of Cognitive Decline for Enrichment of Alzheimer's Disease Clinical Trials. J Prev Alzheimers Dis. 2022;9(3):400-9.

112. Tang L, Wu X, Liu H, Wu F, Song R, Zhang W, et al. Individualized Prediction of Early Alzheimer's Disease Based on Magnetic Resonance Imaging Radiomics, Clinical, and Laboratory Examinations: A 60-Month Follow-Up Study. J Magn Reson Imaging. 2021;54(5):1647-57.

113. Ten Kate M, Redolfi A, Peira E, Bos I, Vos SJ, Vandenberghe R, et al. MRI predictors of amyloid pathology: results from the EMIF-AD Multimodal Biomarker Discovery study. Alzheimers Res Ther. 2018;10(1):100.

114. Teng L, Li Y, Zhao Y, Hu T, Zhang Z, Yao Z, Hu B. Predicting MCI progression with FDG-PET and cognitive scores: a longitudinal study. BMC Neurol. 2020;20(1):148.

115. Thung KH, Yap PT, Ehsan A, Whan LS, Dinggang S. Conversion and time-to-conversion predictions of mild cognitive impairment using low-rank affinity pursuit denoising and matrix completion. Med Image Anal. 2018;45:68-82.

116. Tolonen A, Rhodius-Meester HFM, Bruun M, Koikkalainen J, Barkhof F, Lemstra AW, et al. Data-Driven Differential Diagnosis of Dementia Using Multiclass Disease State Index Classifier. Front Aging Neurosci. 2018;10:111.

117. Varatharajah Y, Ramanan VK, Iyer R, Vemuri P. Predicting Short-term MCI-to-AD Progression Using Imaging, CSF, Genetic Factors, Cognitive Resilience, and Demographics. Sci Rep. 2019;9(1):2235.

118. Vecchio F, Miraglia F, Iberite F, Lacidogna G, Guglielmi V, Marra C, et al. Sustainable method for Alzheimer dementia prediction in mild cognitive impairment: Electroencephalographic connectivity and graph theory combined with apolipoprotein E. Ann Neurol. 2018;84(2):302-14.

119. Velazquez M, Lee Y. Random forest model for feature-based Alzheimer's disease conversion prediction from early mild cognitive impairment subjects. PLoS One. 2021;16(4):e0244773.

120. Velazquez M, Lee Y. Multimodal ensemble model for Alzheimer's disease conversion prediction from Early Mild Cognitive Impairment subjects. Comput Biol Med. 2022;151(Pt A):106201.

121. Westman E, Muehlboeck JS, Simmons A. Combining MRI and CSF measures for classification of Alzheimer's disease and prediction of mild cognitive impairment conversion. Neuroimage. 2012;62(1):229-38.

122. Willette AA, Calhoun VD, Egan JM, Kapogiannis D. Prognostic classification of mild cognitive impairment and Alzheimer's disease: MRI independent component analysis. Psychiatry Res. 2014;224(2):81-8.

123. Wu Y, Wang X, Gu C, Zhu J, Fang Y. Investigating predictors of progression from mild cognitive impairment to Alzheimer’s disease based on different time intervals. Age Ageing. 2023;52(9):afad182.

124. Yao D, Calhoun VD, Fu Z, Du Y, Sui J. An ensemble learning system for a 4-way classification of Alzheimer’s disease and mild cognitive impairment. J Neurosci Methods. 2018;302:75-81.

125. Ye J, Farnum M, Yang E, Verbeeck R, Lobanov V, Raghavan N, et al. Sparse learning and stability selection for predicting MCI to AD conversion using baseline ADNI data. BMC Neurol. 2012;12:46.

126. Yi F, Yang H, Chen D, Qin Y, Han H, Cui J, et al. XGBoost-SHAP-based interpretable diagnostic framework for alzheimer’s disease. BMC Med Inform Decis Mak. 2023;23(1):137.

127. Youn YC, Kim HR, Shin HW, Jeong HB, Han SW, Pyun JM, et al. Prediction of amyloid PET positivity via machine learning algorithms trained with EDTA-based blood amyloid-β oligomerization data. BMC Med Inform Decis Mak. 2022;22(1):286.

128. Young J, Modat M, Cardoso MJ, Mendelson A, Cash D, Ourselin S. Accurate multimodal probabilistic prediction of conversion to Alzheimer's disease in patients with mild cognitive impairment. Neuroimage Clin. 2013;2:735-45.

129. Yue L, Hu D, Zhang H, Wen J, Wu Y, Li W, et al. Prediction of 7-year's conversion from subjective cognitive decline to mild cognitive impairment. Hum Brain Mapp. 2021;42(1):192-203.

130. Zandifar A, Fonov VS, Ducharme S, Belleville S, Collins DL. MRI and cognitive scores complement each other to accurately predict Alzheimer's dementia 2 to 7 years before clinical onset. Neuroimage Clin. 2020;25:102121.

131. Zhang D, Shen D. Multi-modal multi-task learning for joint prediction of multiple regression and classification variables in Alzheimer's disease. Neuroimage. 2012;59(2):895-907.

132. Zhang D, Shen D. Predicting future clinical changes of MCI patients using longitudinal and multimodal biomarkers. PLoS One. 2012;7(3):e33182.

133. Zhang D, Wang Y, Zhou L, Yuan H, Shen D. Multimodal classification of Alzheimer's disease and mild cognitive impairment. Neuroimage. 2011;55(3):856-67.

134. Zhang J, He X, Liu Y, Cai Q, Chen H, Qing L. Multi-modal cross-attention network for Alzheimer's disease diagnosis with multi-modality data. Comput Biol Med. 2023;162:107050.

135. Zhang Y, Ghose U, Buckley NJ, Engelborghs S, Sleegers K, Frisoni GB, et al. Predicting AT(N) pathologies in Alzheimer's disease from blood-based proteomic data using neural networks. Front Aging Neurosci. 2022;14:1040001.

136. Zhang Z, Huang H, Shen D. Integrative analysis of multi-dimensional imaging genomics data for Alzheimer's disease prediction. Front Aging Neurosci. 2014;6:260.

137. Zhao X, Sui H, Yan C, Zhang M, Song H, Liu X, Yang J. Machine-Based Learning Shifting to Prediction Model of Deteriorative MCI Due to Alzheimer’s Disease - A Two-Year Follow-Up Investigation. Curr Alzheimer Res. 2022;19(10):708-15.

138. Zheng Y, Liu Y, Wu J, Xie Y, Yang S, Li W, et al. Predicted Cognitive Conversion in Guiding Early Decision-Tailoring on Patients With Cognitive Impairment. Front Aging Neurosci. 2021;13:813923.
