# Supplementary material for: Diagnostic and prognostic multimodal prediction models in Alzheimer's disease: A scoping review
Source: J Alzheimers Dis. 2025 Jun 26;108(1 Suppl):S209–21. doi: 10.1177/13872877251351630 (PMC12583647; doi:10.1177/13872877251351630)
Supplement: sj-docx-4-alz-10.1177_13872877251351630 - Supplemental material for Diagnostic and prognostic multimodal prediction models in Alzheimer's disease: A scoping review [file sj-docx-4-alz-10.1177_13872877251351630.docx]

**
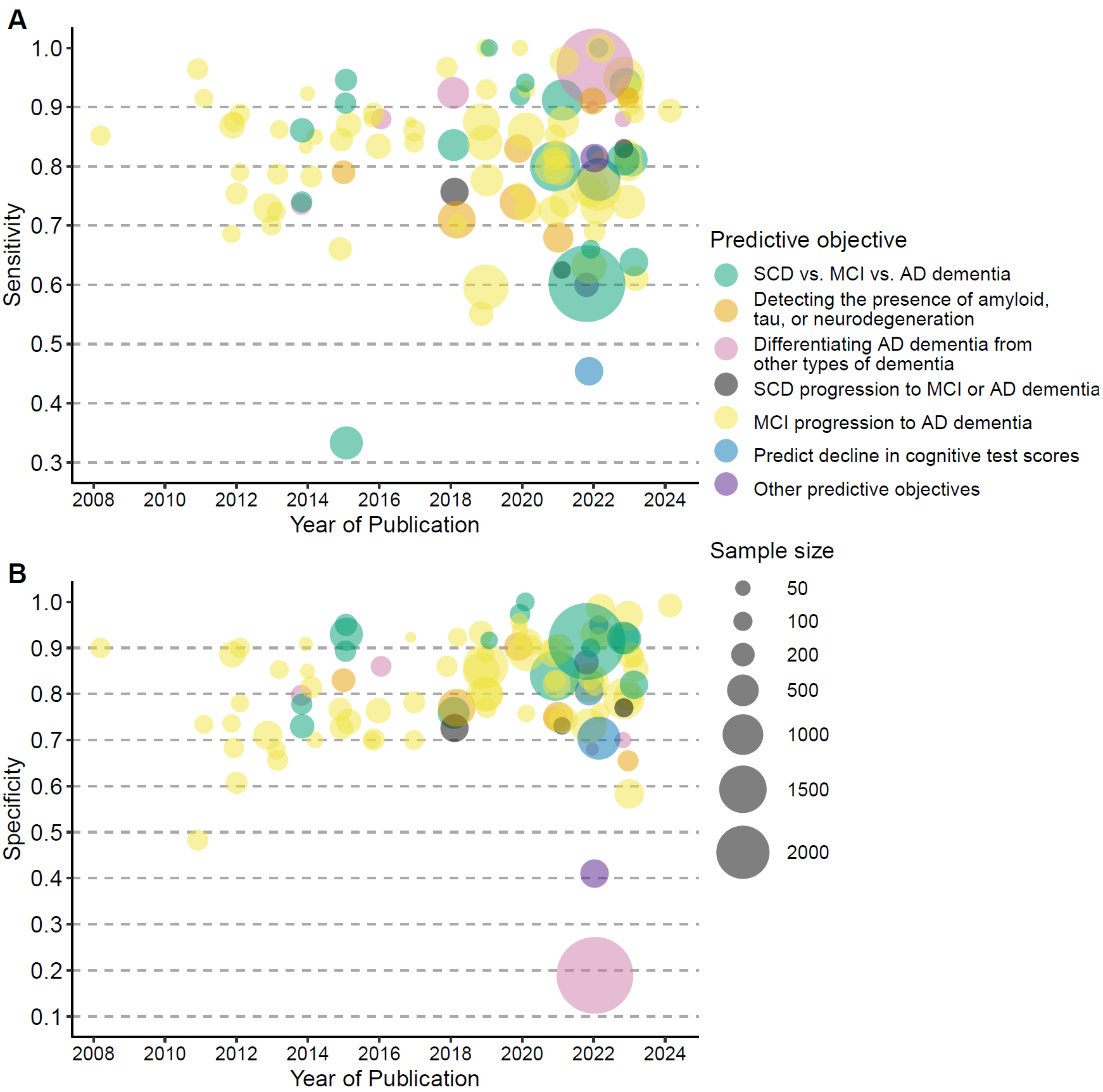
**

**Supplemental Figure 1. Sensitivity and specificity of prediction models**

**(A) Sensitivity of the prediction models. (B) Specificity of the prediction models.**

AD: Alzheimer’s disease; MCI: mild cognitive impairment; SCD: subjective cognitive decline.


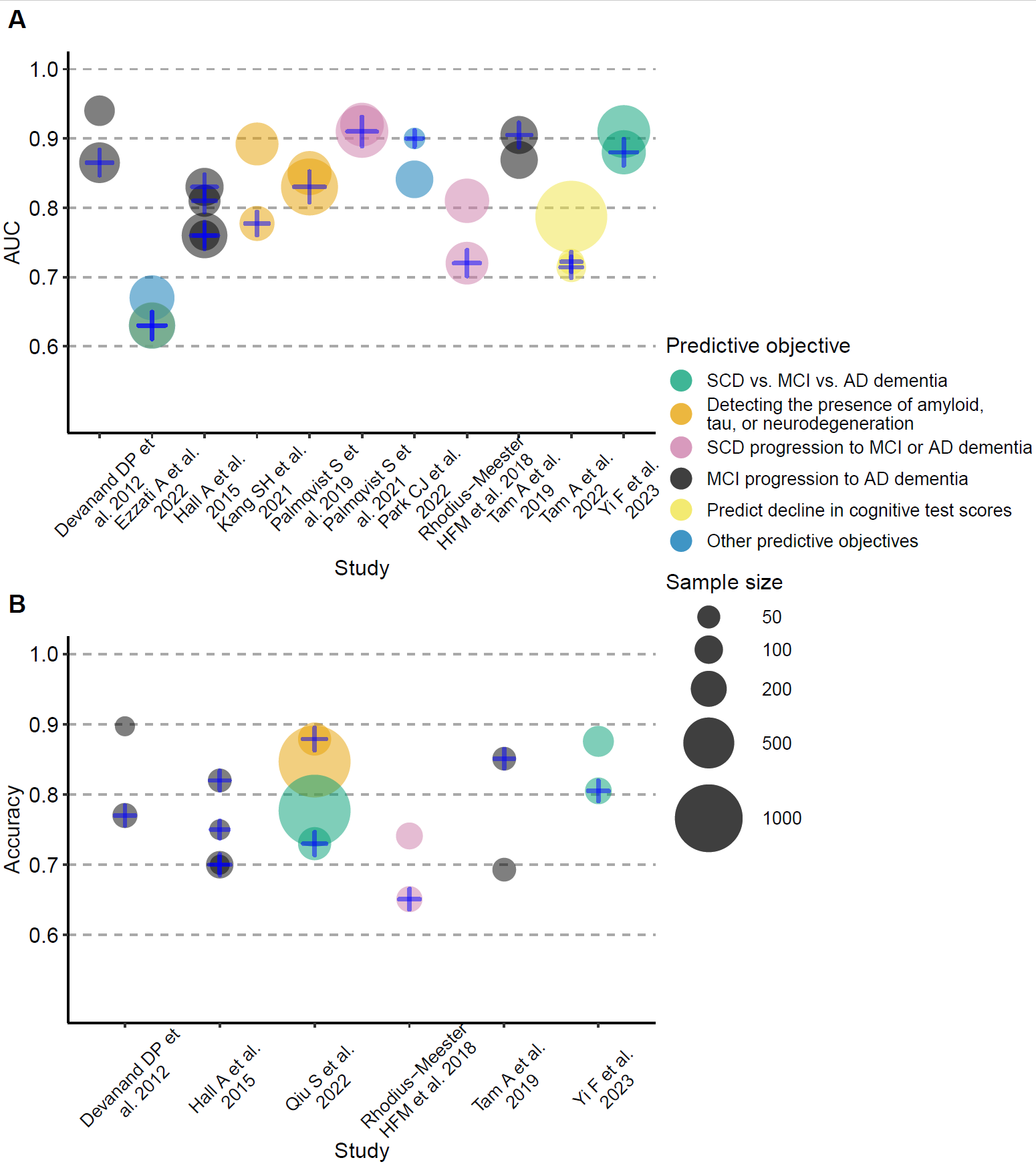


**Supplemental Figure 2. Performance of prediction models in development and external validation datasets**

**(A) AUC of the prediction models. (B) Accuracy of the prediction models.**

AD: Alzheimer’s disease; AUC: Area under the Receiver Operating Characteristic Curve; MCI: mild cognitive impairment, SCD: subjective cognitive decline.

Note: The plus markers in the figure indicate the prediction models that have undergone external validations.
