# Supplementary material for: Diagnostic and prognostic multimodal prediction models in Alzheimer's disease: A scoping review
Source: J Alzheimers Dis. 2025 Jun 26;108(1 Suppl):S209–21. doi: 10.1177/13872877251351630 (PMC12583647; doi:10.1177/13872877251351630)
Supplement: sj-docx-5-alz-10.1177_13872877251351630 - Supplemental material for Diagnostic and prognostic multimodal prediction models in Alzheimer's disease: A scoping review [file sj-docx-5-alz-10.1177_13872877251351630.docx]

**Supplemental Table 1. The associations between the year of publication and sample size, AUC, and accuracy**

|  | **Model 1** | | **Model 2** | |
| --- | --- | --- | --- | --- |
|  | **β-coefficient for year of publication (95% CI)** | **p** | **β-coefficient for year of publication (95% CI)** | **p** |
| **Sample size** | 42 (15-68) | 0.002 | 42 (15-68) | 0.002 |
| **AUC** | 0.007 (0.001-0.012) | 0.032 | 0.007 (0.001-0.012) | 0.029 |
| **Accuracy** | 0.005 (0.001-0.009) | 0.020 | 0.005 (0.001-0.009) | 0.014 |

AUC: Area under the curve.

Note: The results are from linear regressions with robust standard errors, controlling for predictive objectives of the studies for Model 1 and additionally controlling for whether the study is based on ADNI for Model 2.
